# Structural MRI signature predicts tau staging in Alzheimer's disease

**DOI:** 10.1002/dad2.70451

**Published:** 2026-08-26

**Authors:** Martina Pulze, Sara Garbarino, Luigi Lorenzini, Alessio Cirone, Shahzad Ali, Lorenzo Gualco, Federico Massa, Dario Arnaldi, Beatrice Orso, Mattia Losa, Wendy Kreshpa, Stefano Caneva, Matteo Parodi, Luca Sofia, Stefano Raffa, Gianmario Sambuceti, Massimo Caulo, Emanuele Pravatà, Fabrizio Levrero, Marco Bozzali, Silvia Morbelli, Michele Piana, Antonio Uccelli, Luca Roccatagliata, Andrea Chincarini, Matteo Pardini, Alzheimer's Disease Neuroimaging Initiative

**Affiliations:** ^1^ Department of Neuroscience, Rehabilitation, Ophthalmology, Genetics, Maternal and Child Health (DINOGMI) University of Genoa Genoa Italy; ^2^ Department of Mathematics (DIMA) University of Genoa Genoa Italy; ^3^ IRCCS Azienda Ospedaliera Metropolitana Genoa Italy; ^4^ Department of Radiology and Nuclear Medicine Amsterdam University Medical Centre, Vrije Universiteit Amsterdam Amsterdam The Netherlands; ^5^ Department of Pharmacy and Biotechnology Alma Mater Studiorum ‐ University of Bologna Bologna Italy; ^6^ SS Antonio e Biagio e C. Arrigo University Hospital Alessandria Italy; ^7^ Department of Health Sciences University of Genoa Genoa Italy; ^8^ Department of Neuroscience, Imaging and Clinical Sciences University G. d'Annunzio of Chieti‐Pescara Chieti Italy; ^9^ Rita Levi Montalcini's Department of Neuroscience University of Torino Turin Italy; ^10^ AOU Città Della Salute e Della Scienza di Torino Turin Italy; ^11^ Department of Medical Sciences University of Turin Turin Italy; ^12^ National Institute for Nuclear Physics (INFN) Genoa Italy

**Keywords:** Alzheimer's disease, atrophy, MRI, tau

## Abstract

**INTRODUCTION:**

Tau positron emission tomography (PET) probes Alzheimer's disease (AD) severity via regional tau spread but is not widely available. We tested whether multiregion structural magnetic resonance imaging (MRI) could approximate individual tau burden.

**METHODS:**

We studied 378 Alzheimer's Disease Neuroimaging Initiative (ADNI) participants with mild cognitive impairment (MCI)‐AD or AD dementia with paired T1‐MRI and [18F]flortaucipir tau‐PET (≤6 months apart). Regional cortical thickness and volume were extracted with FreeSurfer. Principal component analysis and multivariable linear regression yielded MRI signatures of tau‐PET standardized uptake value ratio (SUVR) in Braak composite regions (I, III–IV, V–VI), a meta‐temporal region of interest (ROI), and a global neocortical meta‐ROI. Performance and high/low tau classification were evaluated by leave‐one‐out cross‐validation against published cut‐offs.

**RESULTS:**

MRI signatures were significantly associated with tau‐PET burden across all regions (*p* < 0.001). High‐versus‐low tau discrimination varied: AUC ≈ 0.70 in Braak I, ≈0.89 in Braak V–VI, and ≈0.90 globally.

**Discussion:**

Here we provide proof‐of‐concept evidence that multiregion T1‐MRI patterns can inform on tau‐PET burden in AD and may support approximate tau staging when tau‐PET is unavailable, especially in subjects with more advanced tau burden.

## Highlights

- Tau PET is used as a staging marker in AD, but it is not widely available.
- Here we evaluated whether T1w MRI could be used as a surrogate staging marker in AD.
- Composite MRI metrics were able to discriminate across tau burden levels.

## 1 BACKGROUND

The latest criteria for diagnosing Alzheimer's disease (AD) incorporate imaging and fluid biomarkers that capture AD pathology in vivo, aiming for a biological definition of the disease.^1^, ^2^ The most recent criteria^1^ also provide guidelines for staging AD biological severity based on cortical tau regional spread, leveraging the topographic and quantitative information of positron emission tomography (PET) biomarkers in a four‐category staging system. This staging approach has been shown to be relevant for guiding patient selection in recent clinical trials for anti‐amyloid disease‐modifying treatments.^3^ However, the limited availability of tau‐PET in routine clinical practice underscores the need for widely accessible surrogate markers capable of approximating tau‐PET findings in patients with biologically confirmed AD. Moreover, the development of a surrogate approach based on widely available imaging modalities such as magnetic resonance imaging (MRI) could allow retrospective analyses of already acquired data, including valuable longitudinal cohorts and clinical trials.

In neuropathological studies, neurofibrillary tangles are known to be spatially correlated with the distribution of gray matter loss in AD.^4^ For example, Whitwell and colleagues showed that MRI gray matter loss topographically mirrored Braak stages of tau pathology at autopsy, validating structural MRI as an approximate in vivo surrogate for tau spread.^5^ Building upon this neuropathological evidence, several studies have investigated the in vivo association between regional atrophy, assessed through structural MRI, and co‐localized tau‐PET uptake values. These studies have provided valuable insights into the temporal dynamics of regional tau accrual in AD while highlighting tau‐PET as a potential prognostic marker for both atrophy progression^6^ and clinical decline.^7^

From a clinical perspective, MRI is widely available, non‐invasive, and usually the first neuroimaging technique used in the work‐up of neurodegenerative conditions, while tau‐PET is constrained by cost, limited availability, and radioprotection issues. Although the association between spatial tau burden and atrophy is well documented,^8^ few studies have examined whether MRI‐derived signatures can reliably predict categorical tau staging. Dallaire‐Théroux and colleagues^9^ showed that *ante mortem* T1 MRI data could predict *post mortem* Braak tangle stage with ∼62% accuracy, while Karlsson and collaborators^10^ employed machine learning on multimodal data (including MRI) to estimate tau‐PET load. Together, these initial findings suggest that MRI measures could complement or even reduce the need for tau‐PET in clinical settings, but the potential of clinically available MRI to serve as a surrogate marker of tau burden and stage AD patients remains underexplored. Importantly, throughout this work, such a MRI‐based surrogate is conceived strictly as a tau‐staging aid to be applied within a biomarker‐confirmed AD population and not as a means of diagnosing AD from MRI alone or of estimating tau burden in other neurodegenerative conditions.

In this context, a key consideration is whether MRI metrics derived from T1‐weighted images can provide sufficient information to infer the severity and distribution of tau pathology in subjects with AD, given the widespread availability of volumetric T1‐weighted imaging in the context of neurodegenerative disorders. To address this question, we explored whether a data‐driven combination of regional T1‐weighted MRI gray matter atrophy measures – referred to as a MRI tau signature – could determine the likelihood of an individual AD patient of having high or low tau levels at the time of the scan. This approach would allow patients to be classified into discrete tau staging categories based on a simple MRI scan. It is important to underscore that the proposed approach is intended exclusively for use in subjects with a biomarker‐confirmed diagnosis of AD: It is designed as a tau staging tool within the AD population and not meant to support a diagnosis of AD from MRI alone, nor to estimate tau burden in other neurodegenerative conditions. If successful, such a strategy could optimize resource allocation by prioritizing tau‐PET imaging for individuals in the intermediate tau spectrum. It could also improve the screening process for clinical trials and potentially provide a non‐invasive alternative for monitoring longitudinal tau burden in AD patients.

To this end, we used paired Alzheimer's Disease Neuroimaging Initiative (ADNI) tau‐PET and T1‐weighted MRI data to (1) derive data‐driven, multiregion MRI‐based tau signatures by combining weighted regional cortical thickness measures into composite scores and (2) evaluate whether these signatures can stratify individuals with AD as having high versus low tau burden across Braak stage–based composite regions and at the whole‐brain level.

## 2 METHODS

### 2.1 Dataset and subject selection

Data were obtained from the ADNI database (https://adni.loni.usc.edu/). We selected participants with available AV1451‐PET brain imaging data selected according to the following inclusion criteria: a diagnosis of mild cognitive impairment (MCI) due to AD or AD dementia, the availability of volumetric T1 images of sufficient quality for allowing processing with FreeSurfer (version 7.1.1) and extraction of cortical measures based on the Desikan–Killiany (DK) atlas,^11^ and a time range of no more than 6 months between the acquisition of MRI and tau‐PET.

**RESEARCH IN CONTEXT**

1. **Systematic review**: The authors reviewed the literature using PubMed and Google Scholar. Tau‐PET imaging is currently the best‐characterized biomarker for the biological staging of disease severity in AD; however, access is currently limited. While several studies explored the potential of tau‐PET to predict longitudinal atrophy, the possibility of using structural MRI to predict tau burden, at least categorically, has not been thoroughly explored.
2. **Interpretation**: A composite MRI metric based on weighted regional thickness was able to predict and classify subjects as having high or low tau burden across Braak regions and the whole brain.
3. **Future directions**: Validation of the proposed approach in an independent population is warranted to confirm its reliability.

The final dataset included 378 subjects (mean age: 72.1 ± 0.4 years; 58% males), of which 273 had MCI (mean age: 71.5 ± 0.4 years; 57.5% males) and 105 subjects had dementia (mean age: 73.7 ± 0.8, 59% males).

### 2.2 Imaging metrics

#### 2.2.1 MRI acquisition and processing

T1‐weighted (T1w) brain MRI image scans were acquired and preprocessed according to the ADNI protocol, as previously described (https://adni.loni.usc.edu/). FreeSurfer version 7.1.1 was used to compute regional gray matter metrics (cortical thickness as primary outcome metrics and volumes as secondary output) from T1w MRI images within gray matter regions of interest (ROIs) according to the DK atlas.^11^ The MRI closest to the available tau‐PET scans were used for subjects with multiple MRI time points.

#### 2.2.2 PET acquisition and processing

[18F]flortaucipir PET scans were acquired and preprocessed according to the ADNI protocol (https://adni.loni.usc.edu/methods/pet‐analysis‐method/pet‐analysis/), as previously described.^12^ Cerebellum‐normalized regional [18F]flortaucipir PET data were already available on file from the ADNI repository. For subjects with multiple tau‐PET acquisitions, only the first available scan was used for all analyses.

Tau burden was assessed using standardized uptake value ratios (SUVRs) from composite ROIs. Specifically, we computed average tau SUVR values in three areas according to the Braak staging model,^13^ i.e., Braak I region, Braak III–IV regions, and Braak V–VI regions. Furthermore, we computed the average SUVR for the meta‐temporal ROI. A detailed description of the DK regions included in the Braak stage region and the meta‐temporal ROI is reported in Table S1. The SUVRs were already computed and available in the ADNI repository based on the regional, volume‐weighted SUVRs as previously described.^14^ Finally, a global tau SUVR was computed using a meta‐ROI encompassing the frontal, parietal, and lateral temporal regions, using the same approach as in the registrative clinical trials for donanemab.^3^

### 2.3 Statistical analysis

**FIGURE 1:**
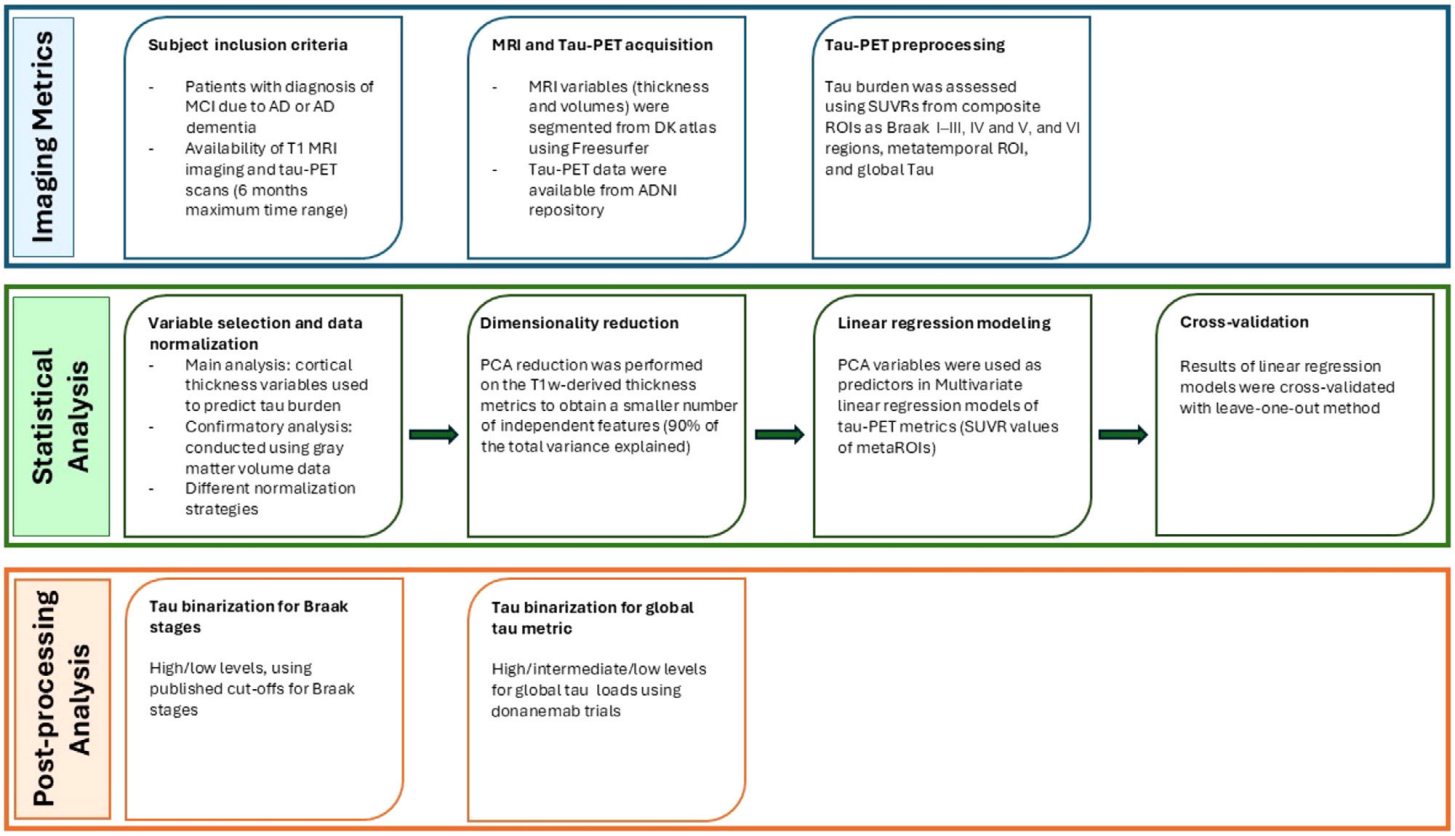
Flow chart summarizing pipeline of study, including imaging acquisition, data preprocessing, dimensionality reduction, and statistical modeling for prediction of tau burden from MRI‐derived metrics.

The overall pipeline for the main analysis is summarized in Figure 1 and detailed below. Statistical models were employed to identify the combination of T1‐based MRI regional measures that best correlates with tau‐PET measures. This manuscript will collectively refer to the resulting combinations of MRI regional measures as “tau signatures” (i.e., tau signature for Braak regions, meta‐temporal ROI, and global tau).

#### 2.3.1 Variable selection and data normalization

First, we focused on cortical thickness as our key MRI measure to predict tau burden, as it has been shown to be more sensitive to age‐related neurodegeneration and more accurate in predicting tau pathology compared to volumetric measures.^15^ This choice also rests on clear structural and pathological considerations: Cortical thickness is a direct readout of the integrity of the cortical ribbon, in which the neuronal, dendritic, and synaptic loss driven by neurofibrillary tau pathology takes place, and it is expressed in millimeters and largely independent of head size. A confirmatory analysis using gray matter volume is reported in the Supplementary Materials. Different normalization strategies (intracranial volume, global mean and standard deviation, and ROI‐specific z‐score) were explored in validation analyses with no material differences across results (Supplementary Materials).

#### 2.3.2 Dimensionality reduction

To reduce the dimensionality, we performed a principal component analysis (PCA) on the regional T1w‐derived thickness metrics and obtained a smaller set of independent latent features related to patterns of structural variation. This transformation reduced the original set of correlated features (68 regional thickness metrics) into smaller orthogonal components, effectively capturing the significant sources of variance while minimizing redundancy. Components were retained up to 90% of the total variance explained, that is, 21 components of PCA.

#### 2.3.3 Linear regression modeling

These 21 T1w‐derived metrics were then used as predictors in a multivariate linear regression framework to predict tau‐PET metrics. Five different regression models were built, with the five tau‐PET ROI SUVRs as outcome (the three Braak regions, the meta‐temporal ROI, and the global tau SUVR) and the 21 thickness components as predictors. Regression results were computed using leave‐one‐out cross‐validation. Statistical significance was set at *p* < 0.001.

To enable regional interpretation, the resulting regression coefficients were back‐projected from PCA space into the original feature space, deriving the contribution (load) of cortical thickness in each region to the prediction of each tau ROI. For each tau‐PET metric, the sum of all weights represents the MRI signature: a spatially resolved weight map reflecting the contribution of each cortical region to the predicted tracer uptake.

#### 2.3.4 Classification of tau burden

To facilitate the interpretation and description of clinical characteristics of the selected cohort, we stratified participants as having low or high tau burden for each of the Braak stage ROI and for the meta‐temporal ROI using established cut‐offs found in the literature.^16^ We also used the same approach for the global tau ROI, dividing participants into high, intermediate, and low tau burden based on published cut‐offs in disease‐modifying drug trials^3^ (low‐tau SUVR < 1.10, high‐tau SUVR > 1.46). Using this stratification in discrete groups, we computed the odds (likelihood ratios) based on the MRI signatures independently for each Braak stage ROI, for the meta‐temporal ROI and the global tau burden ROI. Lastly, we computed the area under the curve (AUC) values for each model. A leave‐one‐out approach was used to compute corrected AUC values.

### 2.4 Ethics statement

The ADNI protocols were approved by each cohort's respective institutional ethical review board. All participants provided written informed consent. The studies were performed in accordance with the Declaration of Helsinki and its later amendments.

## 3 RESULTS

### 3.1 Study population

The clinical characteristics of the study participants, divided according to Braak stages, are reported in Table 1.

**TABLE 1:** The table reports the clinical characteristics of the study participants divided according to tau burden positivity across Braak stages and over the whole brain.

		**Braak I**		**Braak III–IV**		**Braak V–VI**		**Meta‐temporal ROI**		**Global tau**	
	Overall (*n* = 378)	Negative (*n* = 105)	Positive (*n* = 273)	Negative (*n* = 210)	Positive (*n* = 168)	Negative (*n* = 341)	Positive (*n* = 37)	Negative (*n* = 256)	Positive (*n* = 122)	Low/medium (*n* = 341)	High (*n* = 37)
Age, mean (SD)	72.09 (7.50)	70.56 (8.33)	72.69 (7.08)	71.62 (7.75)	72.68 (7.15)	72.59 (7.38)	67.53 (7.16)	71.90 (7.54)	72.49 (7.44)	72.64 (7.36)	67.09 (6.97)
Sex = male, *N* (%)	219 (57.9)	63 (60.0)	156 (57.1)	133 (63.3)	86 (51.2)	204 (59.8)	15 (40.5)	157 (61.3)	62 (50.8)	205 (60.1)	14 (37.8)
Education, years, mean (SD)	16.03 (2.54)	15.87 (2.69)	16.09 (2.49)	16.19 (2.67)	15.82 (2.37)	16.06 (2.58)	15.68 (2.10)	16.25 (2.59)	15.56 (2.38)	16.07 (2.58)	15.65 (2.10)
MMSE, Mean (SD)	27.00 (2.77)	27.84 (2.10)	26.68 (2.93)	27.74 (2.20)	26.07 (3.12)	27.26 (2.55)	24.57 (3.47)	27.66 (2.25)	25.61 (3.22)	27.26 (2.57)	24.65 (3.42)
MoCA, Mean (SD)	22.11 (4.09)	23.18 (3.40)	21.67 (4.27)	23.19 (3.54)	20.70 (4.34)	22.51 (3.72)	18.49 (5.39)	23.17 (3.40)	19.84 (4.52)	22.54 (3.69)	18.36 (5.37)

### 3.2 Cortical thickness signatures of tau burden

**FIGURE 2:**
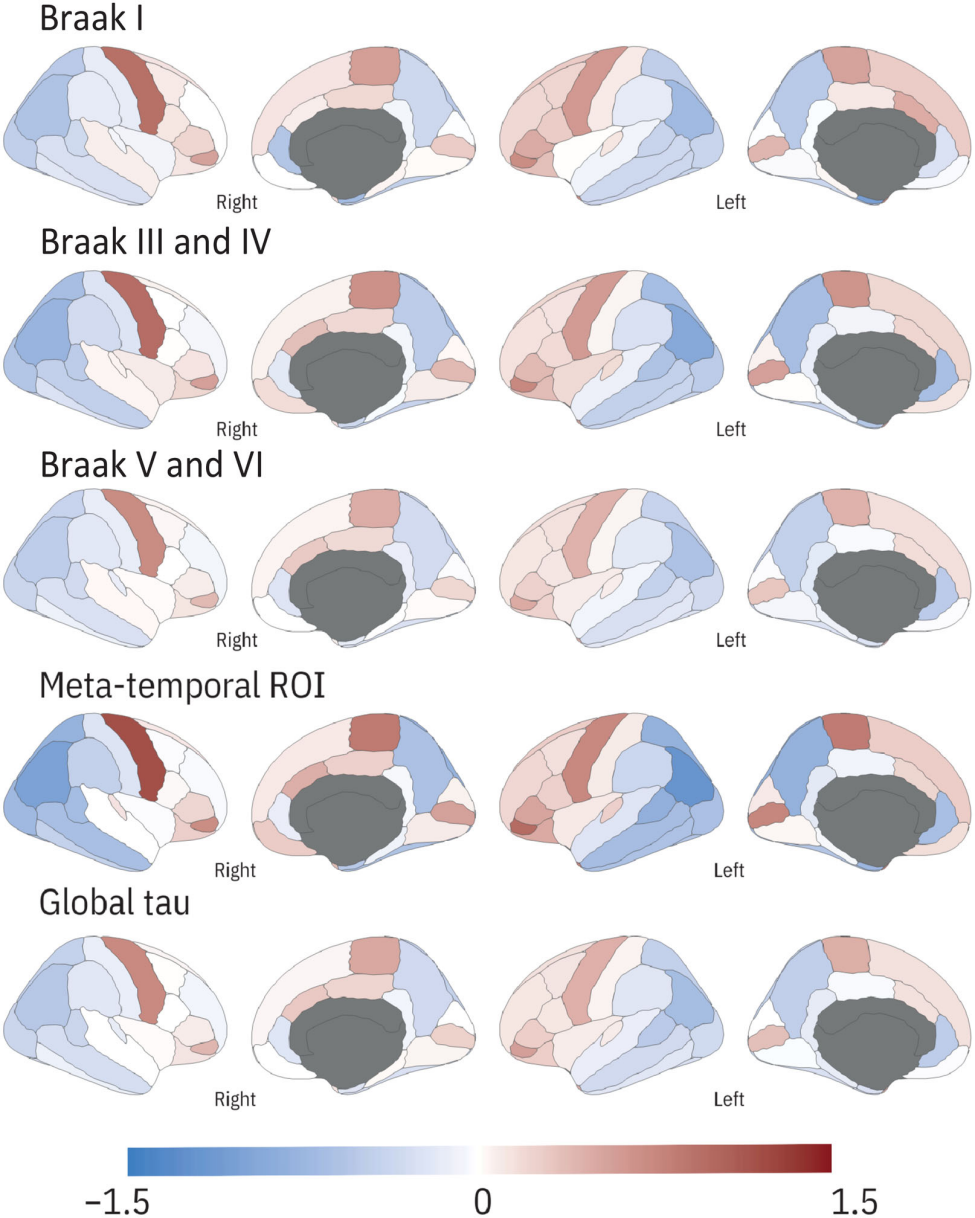
Model coefficients back‐projected on cortical thickness regions correlating with tau burden across each Braak region, meta‐temporal ROI, and global tau. As reported in color bar, shades of blue indicate negative correlations and shades of red positive correlations.

Twenty‐one components from the PCA explained 90% of the total variance in cortical thickness and were used as independent variables in five linear models predicting tau load across the five composite regions. The results of the back‐projection step are shown in Figure 2, which displays the regional coefficients for each cortical region across all five linear models. The coefficients represent the relative weight of each region's cortical thickness in the overall signature. In regions with positive coefficients, higher (i.e., preserved) cortical thickness is associated with a higher likelihood of high tau burden, whereas in regions with negative coefficients, lower (i.e., more damaged) cortical thickness is associated with higher model values (i.e., a higher likelihood of high tau burden). Coefficients are also reported in tree plots in Figures S1, S2, S3, S4, and S5.

Overall, for all models, posterior regions of cortical thickness loss with relatively conserved anterior cortices were associated with a higher likelihood of high tau burden, as expected. The relative relevance of the association of preserved frontal cortices with high tau burden, however, is progressively reduced for higher Braak stages compared to Braak I.

### 3.3 Predictive modeling results

We then evaluated whether the regression models reached an accurate prediction of the outcomes. The cross‐validated coefficient of determination (*R* ^2^) and the associated *p* values of the thickness‐based regression models are reported in Table 2A as a measure of model predictive performance. All models were significant and achieved high values of explained variance. The model predicting tau burden in the Braak I region achieved an *R* ^2^ of 0.47 (*p* < 0.0001), the model for Braak III–IV yielded an *R* ^2^ of 0.56 (*p* < 0.0001). For Braak V–VI, the *R* ^2^ was 0.53 (*p* < 0.0001), and for the meta‐temporal ROI, the model obtained an *R* ^2^ of 0.58 (*p* < 0.0001). Finally, the model predicting global tau burden reported an *R* ^2^ of 0.54 (*p* < 0.0001).

**TABLE 2:** Predictive performance and classification accuracy of MRI signatures for tau‐PET burden.

Panel A. *Linear regression models — R^2^ and p value for each tau‐PET metric*.		
Composite region of interest	*R* ^2^	*p* value
Braak I	0.47	p<0.0001
Braak III–IV	0.56	p<0.0001
Braak V–VI	0.53	p<0.0001
Meta‐temporal ROI	0.58	p<0.0001
Global Tau	0.54	p<0.0001

Panel B. Area under the curve (AUC) values for high versus low tau classification across regions. *p < 0.001 for all AUCs.	
Tau‐PET signature	AUC value*
Braak I	0.70
Braak III–IV	0.72
Braak V–VI	0.89
Meta‐temporal ROI	0.70
Global tau (high vs low)	0.90
Global tau (low vs not low)	0.60
Global tau (high vs not high)	0.89

*Note*: Panel A reports outputs (*R* ^2^ and *p* value) of linear regression models performed using cortical thickness variables for each tau‐PET metric (Braak I, Braak III–IV, Braak V–VI, meta‐temporal ROI and global tau). Panel B reports area under the curve (AUC) values for each Braak meta‐ROI, the meta‐temporal ROI, and the global tau metric, referring to stratification into low‐ and high‐tau burden categories performed in post‐analysis phase. For global tau, stratification includes all pairs of contrasts (high vs low, low vs not low and high vs not high). All AUCs were significant at *p* < 0.001.

### 3.4 Classification of tau burden

**FIGURE 3:**
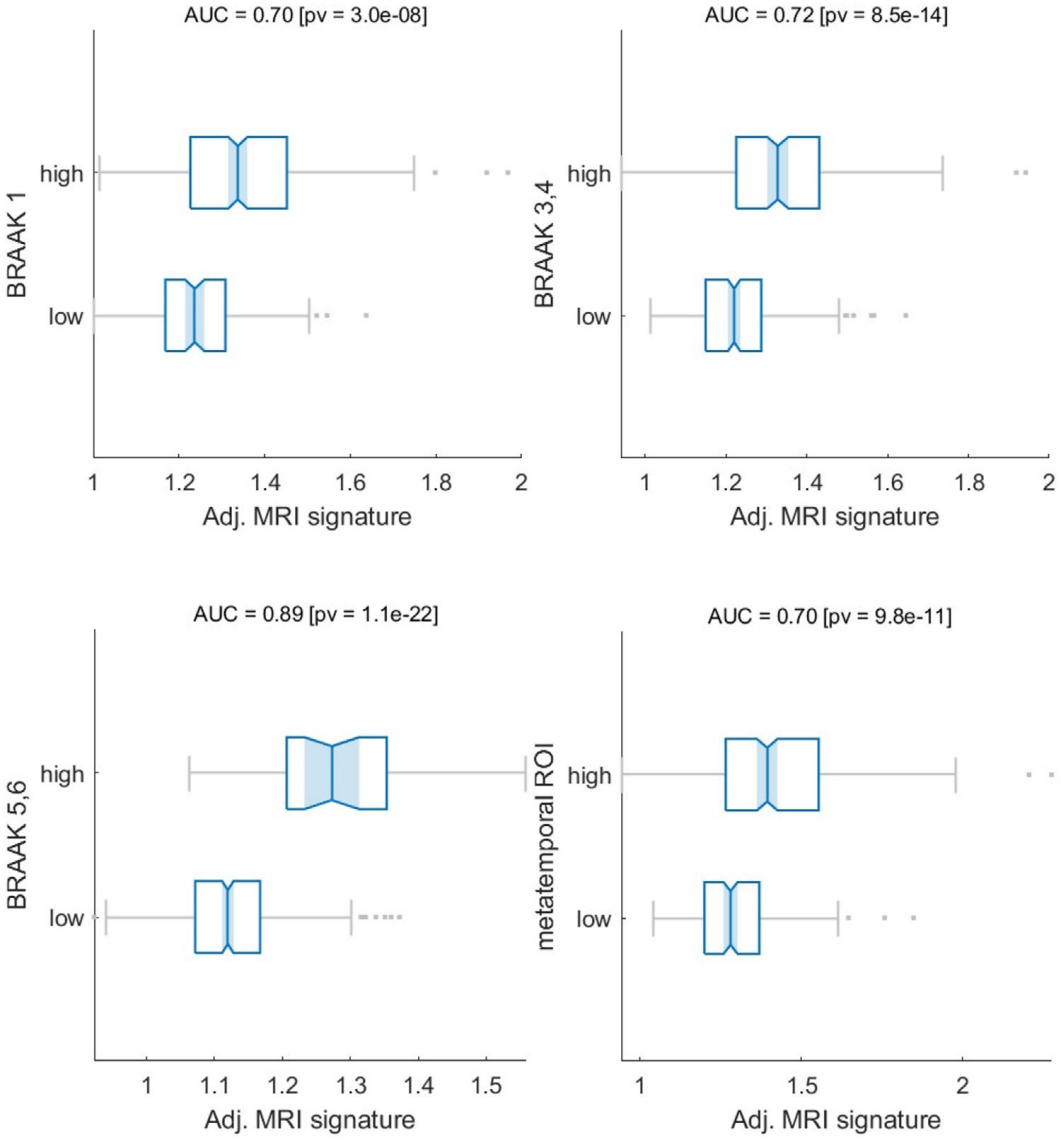
Panels report area under the curve (AUC) and MRI model estimate for each Braak meta‐ROI and meta‐temporal ROI. The line inside each box represents the median, and the top and bottom edges represent the upper and lower quartiles. All statistics (mean and quantiles) are computed over individual data points, each evaluated in cross‐validation with the linear regression model. Whiskers go from the non‐outlier maximum to the non‐outlier minimum. The upper and lower edges of the notch represent an approximation of the 95% confidence interval for the median. *p*v, *p* value.

Figure 3 and Table 2B show the AUC and significance values for the classification in high and low tau burden for each Braak meta‐ROI and the meta‐temporal ROI. All AUCs were highly significant (*p* < 0.001), with the higher AUC (0.89) observed for Braak V–VI. Likelihood ratio plots are reported in Figure S6.

**FIGURE 4:**
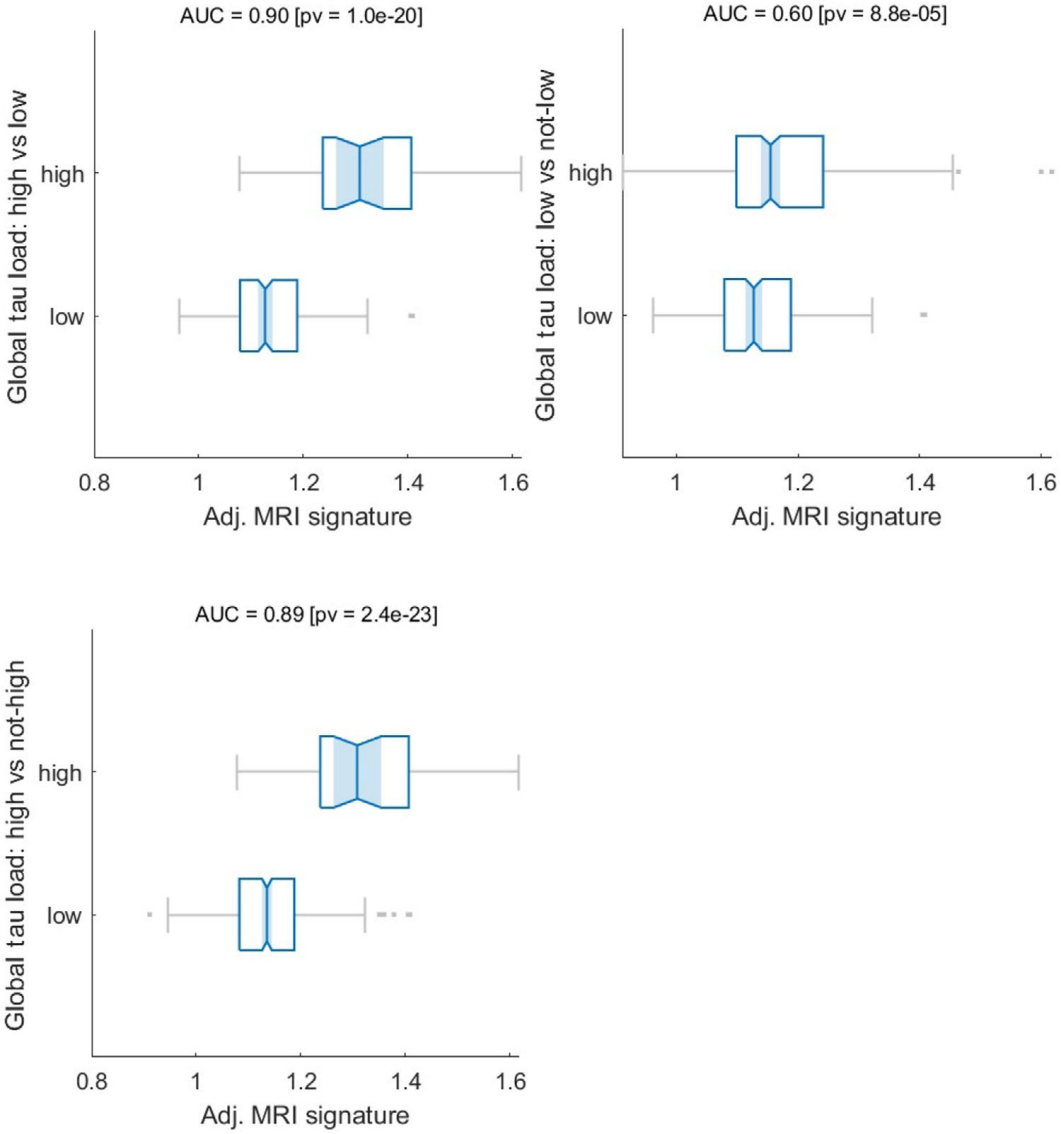
Figure showing MRI model estimate of global tau levels in high‐ and low‐tau burden based on global meta‐ROI SUVR. The line inside each box represents the median, and the top and bottom edges represent the upper and lower quartiles. All statistics (mean and quantiles) are computed over individual data points, each evaluated in cross‐validation with the linear regression model. Whiskers go from the non‐outlier maximum to the non‐outlier minimum. The upper and lower edges of the notch represent an approximation of the 95% confidence interval for the median. *p*v, *p* value.

Figure 4 and Table 2B show the AUCs and significance values for the classification in high, low, and intermediate whole‐brain tau burden based on the global meta‐ROI SUVR. All pairs of contrasts (high vs low, low vs not low, and high vs not high) were highly significant (*p* < 0.001), with the highest AUC (0.9) observed for the high versus low tau burden contrast. Likelihood ratio plots are reported in Figure S7.

For all AUCs, there was no significant difference in the results using different normalization approaches (global mean and standard deviation and individual variable normalization) as described in the supplementary materials (Figures S8, S9, S10, and S11).

### 3.5 Volume‐based results

The use of gray matter volume instead of regional thickness measures retained statistical significance but with lower AUC values, as reported in Table S2. Models based only on demographics and Mini‐Mental State Examination (MMSE) or MoCA scores were not able to predict tau burden in any Braak region, in the meta‐temporal ROI or over the whole brain.

## 4 DISCUSSION

In this study, we developed a composite T1‐weighted MRI metric that classifies AD patients according to regional and global tau‐PET burden. We demonstrated that our T1‐weighted MRI‐based approach could successfully stratify AD individuals into high versus low tau groups across Braak stage regions and a global temporal meta‐ROI using only structural MRI data. These results provide evidence for the potential of a MRI‐based approach, based on widely available structural T1 data, in increasing access to biological disease staging in AD and in allowing for the analysis of already acquired AD data.

We showed that the proposed MRI signatures could categorize individual subjects into high versus low tau categories across the Braak stage ROIs and temporal meta‐ROI, with higher accuracy at more advanced biological stages. This pattern is expected given the temporal lag between initial tau accumulation and ensuing atrophy: Early Braak I–II tau deposition produces subtle structural changes, whereas extensive tau pathology in Braak V–VI is accompanied by more diffuse cortical atrophy.^8^ Importantly, applicability across all Braak stages supports use of our approach not only as a screening tool for subjects with high neocortical tau, but also – albeit with lower accuracy – to identify subjects at the very earliest stages of tau deposition. It should be stressed, however, that the substantially lower discrimination obtained at the earliest Braak stages (AUC ≈ 0.70) meaningfully limits the utility of the approach for reliably detecting the very earliest tau deposition; this specific application should therefore be regarded as exploratory and proof of concept, pending confirmation in independent cohorts.

Our work extends prior research on tau imaging and neurodegeneration. While several studies evaluated the association between regional tau‐PET metrics and gray matter loss, their focus was mainly on baseline tau‐PET in predicting future atrophy^6^, ^17^ rather than the use of volumetric MRI to predict tau burden. Our results shed light on the less explored potential of MRI to predict tau levels, which is not yet included in the proposed staging criteria for AD^1^ and could thus improve the clinical availability of (indirect) tau burden staging in AD patients.

Previous evidence showed regional correlations between tau‐PET SUVR and concomitant gray matter atrophy, with moderate values^6^, ^18^ partly reflecting the temporal lag between regional tau deposition and atrophy.^8^ Compared to such single‐region correlation approaches, our method showed good reliability in classifying AD subjects for tau load, thanks to its focus on binary classification and its integration of data from multiple cortical regions. Multiregion cortical thickness MRI metrics have reliably correlated with AD core biomarkers, outperforming single‐region metrics.^19^ Our binary categorization rather than quantitative SUVR prediction further increased robustness, possibly reducing the impact of focal comorbidities on regional thickness while maintaining the global imprint of tau on AD‐related atrophy.^20^

Our findings extend earlier attempts to use MRI as a tau surrogate. Dallaire‐Théroux et al.^9^ reported that MRI‐derived metrics could classify autopsy Braak stages with ∼62% accuracy, demonstrating proof of concept that structural scans contained a tau signal. We extend this by using in vivo tau‐PET as the reference and achieving substantially higher classification performance, particularly at advanced stages, likely thanks to data‐driven feature integration and contemporaneous MRI–tau pairing. Karlsson et al.^10^ used machine learning on plasma biomarkers, clinical data, and MRI to estimate global tau‐PET load, showing that MRI features contributed even alongside fluid biomarkers. Compared to that work, our unbiased combination of regional thickness data across the whole brain achieved higher accuracies for discrete tau classification. Together, these results support the inclusion of MRI‐based tau surrogates in future iterations of AD staging criteria.

The results on the global tau SUVR confirm those for the Braak ROIs, indicating an excellent discriminatory ability of the proposed MRI‐based approach. This is relevant for future clinical trial design and retrospective analyses of completed trials, where it could support subject selection and provide a longitudinal outcome metric, reducing the need for tau‐PET. In the clinical setting, this approach could help identify subjects with cognitive complaints, structural MRI, and positive blood markers who may benefit from a more detailed work‐up to verify eligibility for a disease‐modifying treatment.

Our approach also allowed exploration of regional associations between cortical thickness and tau burden across disease stages. In interpreting these regional weights it is important to keep in mind that they are the ingredients of a multivariate classifier and not measures of a direct, region‐by‐region biological link: A positive weight in an anterior region does not mean that tau accumulates where the cortex is preserved, but simply that, within the model, relative sparing of anterior cortices alongside posterior thinning helps to recognize the posterior‐predominant atrophy pattern typical of AD. Prefrontal regions showed relative preservation contributing to the tau signature especially at lower Braak stages, in line with disease progression.^21^ Notably, precentral gyrus relative preservation was strongly associated with tau burden in all models, consistent with the resilience of the primary motor cortex to AD pathology. The presence of positive coefficients in some regions does not imply a direct biological relationship between cortical preservation and tau accumulation; rather, they reflect the discriminative logic of a multivariate model capturing the typical posterior‐predominant atrophy gradient of AD, with relative anterior sparing until advanced stages. Thus, when posterior thinning is present, preserved anterior thickness increases the likelihood that the overall pattern is consistent with AD‐related tau pathology.

This study had some limitations that need to be acknowledged.

First, the regional weights for each MRI signature were derived and evaluated within the same ADNI dataset. While leave‐one‐out cross‐validation reduces bias and stability was confirmed across multiple normalization strategies, dataset‐specific effects could not be excluded. ADNI is a well‐characterized research cohort with standardized acquisition and homogeneous participant characteristics; generalization to other populations remains to be established. External validation in independent cohorts – ideally including European or Asian populations, memory clinic samples with heterogeneous protocols, and broader disease severity and comorbidity ranges – is a critical next step before clinical translation. Current results should therefore be interpreted as proof‐of‐concept evidence of the feasibility of MRI‐based tau staging in a controlled research setting, not as definitive performance benchmarks for clinical implementation.

Second, our approach did not explicitly account for comorbidities that could contribute to gray matter loss. Such non‐tau‐related sources of atrophy would be expected to dilute tau‐specific structural patterns and lower AUC; the strong AUC values observed here support the robustness of the underlying MRI–tau signal in this cohort. Performance degradation may occur in clinical populations where comorbidities are more prevalent than in ADNI. More broadly, cortical thickness is a non‐specific marker of neurodegeneration that can be influenced by factors other than tau – including normal aging, cerebrovascular and white‐matter disease, co‐existing proteinopathies such as transactive response DNA‐binding protein 43 or α‐synuclein pathology, and scanner‐ or acquisition‐related variability. In the present work, the regional thickness metrics entered the models after normalization (as detailed in the Methods) but were deliberately not residualized for demographic covariates, so that the resulting signature can be applied directly to individual scans without ancillary demographic modeling; reassuringly, models based on demographics together with global cognition (MMSE or MoCA) alone did not predict tau burden in any region, indicating that the discriminative signal captured by the MRI signatures was not merely a reflection of age, sex, or disease severity.

Third, we relied exclusively on structural T1‐weighted MRI to estimate tau burden. This deliberate choice was intended to maximize accessibility and translatability, particularly in multicenter settings, and to enable the reuse of retrospective datasets including valuable longitudinal cohorts and previously completed clinical trials. However, dependence on a single modality may constrain predictive performance. Future studies should evaluate whether incorporating complementary metrics – such as diffusion MRI, functional MRI, fluid biomarkers or clinical data – into our signature‐based framework can further improve the accuracy and robustness of MRI‐based tau staging. Lastly, while the signature yields subject‐level estimates through fixed regional weights, it should be applied only to populations comparable to the ADNI cohort (biomarker‐confirmed AD with similar inclusion characteristics); it should not be used to diagnose AD from MRI alone or to infer tau burden in other neurodegenerative conditions. The strength of our approach lies in staging known AD patients, and it is not intended for use in subjects without a confirmed diagnosis of AD.

## 5 CONCLUSIONS

Our data point to a novel and robust approach to using widely available cortical thickness data to stratify AD subjects according to tau burden, potentially increasing the equality and appropriateness of access to tau burden assessment across the different settings of care for subjects with AD.

## AUTHOR CONTRIBUTIONS

Drs. Pulze and Pardini designed the study and analyzed the data together with Drs. Garbarino, Lorenzini, and Cirone. Dr. Chincarini supervised the statistical analyses. All authors contributed to the interpretation of data for original content, the draft of the manuscript, and the revision of the text.

## CONFLICT OF INTEREST STATEMENT

M. Pardini reports fees from Novartis, Lilly, Eisai, and Biogen and research support from Novartis and Nutricia. F. Massa received speaker honoraria from Roche Diagnostics S.p.A. and Eli Lilly S.p.A. All other authors have no significant conflict of interest. Author disclosures are available in the Supporting Information.

## CONSENT STATEMENT

The ADNI protocols were approved by each cohort's respective institutional ethical review board. All participants provided written informed consent. The studies were performed in accordance with the Declaration of Helsinki and its later amendments.

## Supporting information



Supporting Information

Supporting Information

## Data Availability

The data set is owned by the Alzheimer's Disease Neuroimaging Initiative (ADNI). Data are publicly and freely available from the http://adni.loni.usc.edu/data‐samples/access‐data/ Institutional Data Access / Ethics Committee (contact via http://adni.loni.usc.edu/data‐samples/access‐data/) upon sending a request that includes the proposed analysis and the named lead investigator.
