# Supplementary material for: Structural MRI signature predicts tau staging in Alzheimer's disease
Source: Alzheimers Dement (Amst). 2026 Aug 26;18(3):e70451. doi: 10.1002/dad2.70451 (PMC13508572; doi:10.1002/dad2.70451)
Supplement: Supplementary file 1 [file DAD2-18-e70451-s001.pdf]

ICMJE Disclosure Forms

Alzheimer's & Dementia: Diagnosis, Assessment & Disease Monitoring (DADM)

Manuscript title: Structural MRI Signature Predicts Tau Staging in Alzheimer's Disease
Manuscript number: DADM-20260508
Corresponding author: Matteo Pardini
Number of authors: 26

Index of authors (one disclosure form each follows):

1. Martina Pulze — None declared
2. Sara Garbarino — None declared
3. Luigi Lorenzini — None declared
4. Alessio Cirone — None declared
5. Shahzad Alì — None declared
6. Lorenzo Gualco — None declared
7. Federico Massa — disclosures reported
8. Dario Arnaldi — None declared
9. Beatrice Orso — None declared
10. Mattia Losa — None declared
11. Wendy Khrespa — None declared
12. Stefano Caneva — None declared
13. Matteo Parodi — None declared
14. Luca Sofia — None declared
15. Stefano Raffa — None declared
16. Gianmario Sambuceti — None declared
17. Massimo Caulo — None declared
18. Emanuele Pravatà — None declared
19. Fabrizio Levrero — None declared
20. Marco Bozzali — None declared
21. Silvia Morbelli — None declared
22. Michele Piana — None declared
23. Antonio Uccelli — None declared
24. Luca Roccatagliata — None declared
25. Andrea Chincarini — None declared
26. Matteo Pardini *(corresponding author)* — disclosures reported

ICMJE Disclosure Form

Date: 2nd July 2026
Your Name: Martina Pulze
Affiliation: *DINOGMI, University of Genoa*
Manuscript Title: Structural MRI Signature Predicts Tau Staging in Alzheimer's Disease
Manuscript Number: DADM-20260508

Relationship / activity / interest	None	Name all entities (or indicate none)	Specifications / comments
**1.** All support for the present manuscript (e.g., funding, provision of study materials, medical writing, article-processing charges, etc.). [No time limit for this item.]	☒	None	—
**2.** Grants or contracts from any entity (if not indicated in item #1 above).	☒	None	—
**3.** Royalties or licenses	☒	None	—
**4.** Consulting fees	☒	None	—
**5.** Payment or honoraria for lectures, presentations, speakers bureaus, manuscript writing or educational events	☒	None	—
**6.** Payment for expert testimony	☒	None	—
**7.** Support for attending meetings and/or travel	☒	None	—
**8.** Patents planned, issued or pending	☒	None	—
**9.** Participation on a Data Safety Monitoring Board or Advisory Board	☒	None	—
**10.** Leadership or fiduciary role in other board, society, committee or advocacy group, paid or unpaid	☒	None	—
**11.** Stock or stock options	☒	None	—
**12.** Receipt of equipment, materials, drugs, medical writing, gifts or other services	☒	None	—
**13.** Other financial or non-financial interests	☒	None	—

☒ I certify that I have answered every question and have not altered the wording of any of the questions on this form.

Signature: Martina Pulze **Date:** 2nd July 2026

ICMJE Disclosure Form

Date: 2nd July 2026

Your Name: Sara Garbarino

Affiliation: *DIMA, University of Genoa; IRCCS Azienda Ospedaliera Metropolitana (Ospedale Policlinico San Martino), Genoa*

Manuscript Title: Structural MRI Signature Predicts Tau Staging in Alzheimer's Disease

Manuscript Number: DADM-20260508

Relationship / activity / interest	None	Name all entities (or indicate none)	Specifications / comments
**1.** All support for the present manuscript (e.g., funding, provision of study materials, medical writing, article-processing charges, etc.). [No time limit for this item.]	☒	None	—
**2.** Grants or contracts from any entity (if not indicated in item #1 above).	☒	None	—
**3.** Royalties or licenses	☒	None	—
**4.** Consulting fees	☒	None	—
**5.** Payment or honoraria for lectures, presentations, speakers bureaus, manuscript writing or educational events	☒	None	—
**6.** Payment for expert testimony	☒	None	—
**7.** Support for attending meetings and/or travel	☒	None	—
**8.** Patents planned, issued or pending	☒	None	—
**9.** Participation on a Data Safety Monitoring Board or Advisory Board	☒	None	—
**10.** Leadership or fiduciary role in other board, society, committee or advocacy group, paid or unpaid	☒	None	—
**11.** Stock or stock options	☒	None	—
**12.** Receipt of equipment, materials, drugs, medical writing, gifts or other services	☒	None	—
**13.** Other financial or non-financial interests	☒	None	—

☒ I certify that I have answered every question and have not altered the wording of any of the questions on this form.

Signature: S. Garbarino **Date:** 2nd July 2026

ICMJE Disclosure Form

Date: 2nd July 2026

Your Name: Luigi Lorenzini

Affiliation: *DINOGMI, University of Genoa; Amsterdam UMC, Vrije Universiteit Amsterdam*

Manuscript Title: Structural MRI Signature Predicts Tau Staging in Alzheimer's Disease

Manuscript Number: DADM-20260508

Relationship / activity / interest	None	Name all entities (or indicate none)	Specifications / comments
**1.** All support for the present manuscript (e.g., funding, provision of study materials, medical writing, article-processing charges, etc.). [No time limit for this item.]	☒	None	—
**2.** Grants or contracts from any entity (if not indicated in item #1 above).	☒	None	—
**3.** Royalties or licenses	☒	None	—
**4.** Consulting fees	☒	None	—
**5.** Payment or honoraria for lectures, presentations, speakers bureaus, manuscript writing or educational events	☒	None	—
**6.** Payment for expert testimony	☒	None	—
**7.** Support for attending meetings and/or travel	☒	None	—
**8.** Patents planned, issued or pending	☒	None	—
**9.** Participation on a Data Safety Monitoring Board or Advisory Board	☒	None	—
**10.** Leadership or fiduciary role in other board, society, committee or advocacy group, paid or unpaid	☒	None	—
**11.** Stock or stock options	☒	None	—
**12.** Receipt of equipment, materials, drugs, medical writing, gifts or other services	☒	None	—
**13.** Other financial or non-financial interests	☒	None	—

☒ I certify that I have answered every question and have not altered the wording of any of the questions on this form.

Signature: Lorenzini **Date:** 2nd July 2026

ICMJE Disclosure Form

Date: 2nd July 2026

Your Name: Alessio Cirone

Affiliation: *IRCCS Azienda Ospedaliera Metropolitana (Ospedale Policlinico San Martino), Genoa*

Manuscript Title: Structural MRI Signature Predicts Tau Staging in Alzheimer's Disease

Manuscript Number: DADM-20260508

Relationship / activity / interest	None	Name all entities (or indicate none)	Specifications / comments
**1.** All support for the present manuscript (e.g., funding, provision of study materials, medical writing, article-processing charges, etc.). [No time limit for this item.]	☒	None	—
**2.** Grants or contracts from any entity (if not indicated in item #1 above).	☒	None	—
**3.** Royalties or licenses	☒	None	—
**4.** Consulting fees	☒	None	—
**5.** Payment or honoraria for lectures, presentations, speakers bureaus, manuscript writing or educational events	☒	None	—
**6.** Payment for expert testimony	☒	None	—
**7.** Support for attending meetings and/or travel	☒	None	—
**8.** Patents planned, issued or pending	☒	None	—
**9.** Participation on a Data Safety Monitoring Board or Advisory Board	☒	None	—
**10.** Leadership or fiduciary role in other board, society, committee or advocacy group, paid or unpaid	☒	None	—
**11.** Stock or stock options	☒	None	—
**12.** Receipt of equipment, materials, drugs, medical writing, gifts or other services	☒	None	—
**13.** Other financial or non-financial interests	☒	None	—

☒ I certify that I have answered every question and have not altered the wording of any of the questions on this form.

Signature: A.C. **Date:** 2nd July 2026

ICMJE Disclosure Form

Date: 2nd July 2026

Your Name: Shahzad Alì

Affiliation: *University of Bologna*

Manuscript Title: Structural MRI Signature Predicts Tau Staging in Alzheimer’s Disease

Manuscript Number: DADM-20260508

Relationship / activity / interest	None	Name all entities (or indicate none)	Specifications / comments
**1.** All support for the present manuscript (e.g., funding, provision of study materials, medical writing, article-processing charges, etc.). [No time limit for this item.]	☒	None	—
**2.** Grants or contracts from any entity (if not indicated in item #1 above).	☒	None	—
**3.** Royalties or licenses	☒	None	—
**4.** Consulting fees	☒	None	—
**5.** Payment or honoraria for lectures, presentations, speakers bureaus, manuscript writing or educational events	☒	None	—
**6.** Payment for expert testimony	☒	None	—
**7.** Support for attending meetings and/or travel	☒	None	—
**8.** Patents planned, issued or pending	☒	None	—
**9.** Participation on a Data Safety Monitoring Board or Advisory Board	☒	None	—
**10.** Leadership or fiduciary role in other board, society, committee or advocacy group, paid or unpaid	☒	None	—
**11.** Stock or stock options	☒	None	—
**12.** Receipt of equipment, materials, drugs, medical writing, gifts or other services	☒	None	—
**13.** Other financial or non-financial interests	☒	None	—

☒ I certify that I have answered every question and have not altered the wording of any of the questions on this form.

Signature: Shahzad Alì **Date:** 2nd July 2026

ICMJE Disclosure Form

Date: 2nd July 2026

Your Name: Lorenzo Gualco

Affiliation: *DINOGMI, University of Genoa; SS Antonio e Biagio e C. Arrigo University Hospital, Alessandria*

Manuscript Title: Structural MRI Signature Predicts Tau Staging in Alzheimer's Disease

Manuscript Number: DADM-20260508

Relationship / activity / interest	None	Name all entities (or indicate none)	Specifications / comments
**1.** All support for the present manuscript (e.g., funding, provision of study materials, medical writing, article-processing charges, etc.). [No time limit for this item.]	☒	None	—
**2.** Grants or contracts from any entity (if not indicated in item #1 above).	☒	None	—
**3.** Royalties or licenses	☒	None	—
**4.** Consulting fees	☒	None	—
**5.** Payment or honoraria for lectures, presentations, speakers bureaus, manuscript writing or educational events	☒	None	—
**6.** Payment for expert testimony	☒	None	—
**7.** Support for attending meetings and/or travel	☒	None	—
**8.** Patents planned, issued or pending	☒	None	—
**9.** Participation on a Data Safety Monitoring Board or Advisory Board	☒	None	—
**10.** Leadership or fiduciary role in other board, society, committee or advocacy group, paid or unpaid	☒	None	—
**11.** Stock or stock options	☒	None	—
**12.** Receipt of equipment, materials, drugs, medical writing, gifts or other services	☒	None	—
**13.** Other financial or non-financial interests	☒	None	—

☒ I certify that I have answered every question and have not altered the wording of any of the questions on this form.

Signature: L. Gualco **Date:** 2nd July 2026

ICMJE Disclosure Form

Date: 2nd July 2026

Your Name: Federico Massa

Affiliation: *DINOGMI, University of Genoa; IRCCS Azienda Ospedaliera Metropolitana (Ospedale Policlinico San Martino), Genoa*

Manuscript Title: Structural MRI Signature Predicts Tau Staging in Alzheimer's Disease

Manuscript Number: DADM-20260508

Relationship / activity / interest	None	Name all entities (or indicate none)	Specifications / comments
**1.** All support for the present manuscript (e.g., funding, provision of study materials, medical writing, article-processing charges, etc.). [No time limit for this item.]	☒	None	—
**2.** Grants or contracts from any entity (if not indicated in item #1 above).	☒	None	—
**3.** Royalties or licenses	☒	None	—
**4.** Consulting fees	☒	None	—
**5.** Payment or honoraria for lectures, presentations, speakers bureaus, manuscript writing or educational events	☐	**Roche Diagnostics S.p.A.; Eli Lilly S.p.A.**	Speaker honoraria.
**6.** Payment for expert testimony	☒	None	—
**7.** Support for attending meetings and/or travel	☒	None	—
**8.** Patents planned, issued or pending	☒	None	—
**9.** Participation on a Data Safety Monitoring Board or Advisory Board	☒	None	—
**10.** Leadership or fiduciary role in other board, society, committee or advocacy group, paid or unpaid	☒	None	—
**11.** Stock or stock options	☒	None	—
**12.** Receipt of equipment, materials, drugs, medical writing, gifts or other services	☒	None	—
**13.** Other financial or non-financial interests	☒	None	—

☒ I certify that I have answered every question and have not altered the wording of any of the questions on this form.

Signature: Massa **Date:** 2nd July 2026

ICMJE Disclosure Form

Date: 2nd July 2026

Your Name: Dario Arnaldi

Affiliation: *DINOGMI, University of Genoa; IRCCS Azienda Ospedaliera Metropolitana (Ospedale Policlinico San Martino), Genoa*

Manuscript Title: Structural MRI Signature Predicts Tau Staging in Alzheimer's Disease

Manuscript Number: DADM-20260508

Relationship / activity / interest	None	Name all entities (or indicate none)	Specifications / comments
**1.** All support for the present manuscript (e.g., funding, provision of study materials, medical writing, article-processing charges, etc.). [No time limit for this item.]	☒	None	—
**2.** Grants or contracts from any entity (if not indicated in item #1 above).	☒	None	—
**3.** Royalties or licenses	☒	None	—
**4.** Consulting fees	☒	None	—
**5.** Payment or honoraria for lectures, presentations, speakers bureaus, manuscript writing or educational events	☒	None	—
**6.** Payment for expert testimony	☒	None	—
**7.** Support for attending meetings and/or travel	☒	None	—
**8.** Patents planned, issued or pending	☒	None	—
**9.** Participation on a Data Safety Monitoring Board or Advisory Board	☒	None	—
**10.** Leadership or fiduciary role in other board, society, committee or advocacy group, paid or unpaid	☒	None	—
**11.** Stock or stock options	☒	None	—
**12.** Receipt of equipment, materials, drugs, medical writing, gifts or other services	☒	None	—
**13.** Other financial or non-financial interests	☒	None	—

☒ I certify that I have answered every question and have not altered the wording of any of the questions on this form.

Signature: D. Arnaldi **Date:** 2nd July 2026

ICMJE Disclosure Form

Date: 2nd July 2026
Your Name: Beatrice Orso
Affiliation: *DINOGMI, University of Genoa*
Manuscript Title: Structural MRI Signature Predicts Tau Staging in Alzheimer's Disease
Manuscript Number: DADM-20260508

Relationship / activity / interest	None	Name all entities (or indicate none)	Specifications / comments
**1.** All support for the present manuscript (e.g., funding, provision of study materials, medical writing, article-processing charges, etc.). [No time limit for this item.]	☒	None	—
**2.** Grants or contracts from any entity (if not indicated in item #1 above).	☒	None	—
**3.** Royalties or licenses	☒	None	—
**4.** Consulting fees	☒	None	—
**5.** Payment or honoraria for lectures, presentations, speakers bureaus, manuscript writing or educational events	☒	None	—
**6.** Payment for expert testimony	☒	None	—
**7.** Support for attending meetings and/or travel	☒	None	—
**8.** Patents planned, issued or pending	☒	None	—
**9.** Participation on a Data Safety Monitoring Board or Advisory Board	☒	None	—
**10.** Leadership or fiduciary role in other board, society, committee or advocacy group, paid or unpaid	☒	None	—
**11.** Stock or stock options	☒	None	—
**12.** Receipt of equipment, materials, drugs, medical writing, gifts or other services	☒	None	—
**13.** Other financial or non-financial interests	☒	None	—

☒ I certify that I have answered every question and have not altered the wording of any of the questions on this form.

Signature: Beatrice Orso **Date:** 2nd July 2026

ICMJE Disclosure Form

Date: 2nd July 2026

Your Name: Mattia Losa

Affiliation: *DINOGMI, University of Genoa*

Manuscript Title: Structural MRI Signature Predicts Tau Staging in Alzheimer's Disease

Manuscript Number: DADM-20260508

Relationship / activity / interest	None	Name all entities (or indicate none)	Specifications / comments
**1.** All support for the present manuscript (e.g., funding, provision of study materials, medical writing, article-processing charges, etc.). [No time limit for this item.]	☒	None	—
**2.** Grants or contracts from any entity (if not indicated in item #1 above).	☒	None	—
**3.** Royalties or licenses	☒	None	—
**4.** Consulting fees	☒	None	—
**5.** Payment or honoraria for lectures, presentations, speakers bureaus, manuscript writing or educational events	☒	None	—
**6.** Payment for expert testimony	☒	None	—
**7.** Support for attending meetings and/or travel	☒	None	—
**8.** Patents planned, issued or pending	☒	None	—
**9.** Participation on a Data Safety Monitoring Board or Advisory Board	☒	None	—
**10.** Leadership or fiduciary role in other board, society, committee or advocacy group, paid or unpaid	☒	None	—
**11.** Stock or stock options	☒	None	—
**12.** Receipt of equipment, materials, drugs, medical writing, gifts or other services	☒	None	—
**13.** Other financial or non-financial interests	☒	None	—

☒ I certify that I have answered every question and have not altered the wording of any of the questions on this form.

Signature: M.L. **Date:** 2nd July 2026

ICMJE Disclosure Form

Date: 2nd July 2026
Your Name: Wendy Khrespa
Affiliation: *DINOGMI, University of Genoa*
Manuscript Title: Structural MRI Signature Predicts Tau Staging in Alzheimer's Disease
Manuscript Number: DADM-20260508

Relationship / activity / interest	None	Name all entities (or indicate none)	Specifications / comments
**1.** All support for the present manuscript (e.g., funding, provision of study materials, medical writing, article-processing charges, etc.). [No time limit for this item.]	☒	None	—
**2.** Grants or contracts from any entity (if not indicated in item #1 above).	☒	None	—
**3.** Royalties or licenses	☒	None	—
**4.** Consulting fees	☒	None	—
**5.** Payment or honoraria for lectures, presentations, speakers bureaus, manuscript writing or educational events	☒	None	—
**6.** Payment for expert testimony	☒	None	—
**7.** Support for attending meetings and/or travel	☒	None	—
**8.** Patents planned, issued or pending	☒	None	—
**9.** Participation on a Data Safety Monitoring Board or Advisory Board	☒	None	—
**10.** Leadership or fiduciary role in other board, society, committee or advocacy group, paid or unpaid	☒	None	—
**11.** Stock or stock options	☒	None	—
**12.** Receipt of equipment, materials, drugs, medical writing, gifts or other services	☒	None	—
**13.** Other financial or non-financial interests	☒	None	—

☒ I certify that I have answered every question and have not altered the wording of any of the questions on this form.

Signature: W. Khrespa **Date:** 2nd July 2026

ICMJE Disclosure Form

Date: 2nd July 2026
Your Name: Stefano Caneva
Affiliation: *DINOGMI, University of Genoa*
Manuscript Title: Structural MRI Signature Predicts Tau Staging in Alzheimer's Disease
Manuscript Number: DADM-20260508

Relationship / activity / interest	None	Name all entities (or indicate none)	Specifications / comments
**1.** All support for the present manuscript (e.g., funding, provision of study materials, medical writing, article-processing charges, etc.). [No time limit for this item.]	☒	None	—
**2.** Grants or contracts from any entity (if not indicated in item #1 above).	☒	None	—
**3.** Royalties or licenses	☒	None	—
**4.** Consulting fees	☒	None	—
**5.** Payment or honoraria for lectures, presentations, speakers bureaus, manuscript writing or educational events	☒	None	—
**6.** Payment for expert testimony	☒	None	—
**7.** Support for attending meetings and/or travel	☒	None	—
**8.** Patents planned, issued or pending	☒	None	—
**9.** Participation on a Data Safety Monitoring Board or Advisory Board	☒	None	—
**10.** Leadership or fiduciary role in other board, society, committee or advocacy group, paid or unpaid	☒	None	—
**11.** Stock or stock options	☒	None	—
**12.** Receipt of equipment, materials, drugs, medical writing, gifts or other services	☒	None	—
**13.** Other financial or non-financial interests	☒	None	—

☒ I certify that I have answered every question and have not altered the wording of any of the questions on this form.

Signature: 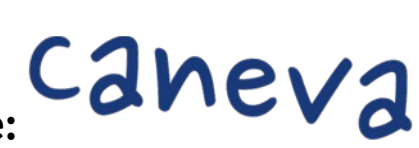

Date: 2nd July 2026

ICMJE Disclosure Form

Date: 2nd July 2026

Your Name: Matteo Parodi

Affiliation: *DINOGMI*, *University of Genoa*

Manuscript Title: Structural MRI Signature Predicts Tau Staging in Alzheimer's Disease

Manuscript Number: DADM-20260508

Relationship / activity / interest	None	Name all entities (or indicate none)	Specifications / comments
**1.** All support for the present manuscript (e.g., funding, provision of study materials, medical writing, article-processing charges, etc.). [No time limit for this item.]	☒	None	—
**2.** Grants or contracts from any entity (if not indicated in item #1 above).	☒	None	—
**3.** Royalties or licenses	☒	None	—
**4.** Consulting fees	☒	None	—
**5.** Payment or honoraria for lectures, presentations, speakers bureaus, manuscript writing or educational events	☒	None	—
**6.** Payment for expert testimony	☒	None	—
**7.** Support for attending meetings and/or travel	☒	None	—
**8.** Patents planned, issued or pending	☒	None	—
**9.** Participation on a Data Safety Monitoring Board or Advisory Board	☒	None	—
**10.** Leadership or fiduciary role in other board, society, committee or advocacy group, paid or unpaid	☒	None	—
**11.** Stock or stock options	☒	None	—
**12.** Receipt of equipment, materials, drugs, medical writing, gifts or other services	☒	None	—
**13.** Other financial or non-financial interests	☒	None	—

☒ I certify that I have answered every question and have not altered the wording of any of the questions on this form.

Signature: Matteo Parodi **Date:** 2nd July 2026

ICMJE Disclosure Form

Date: 2nd July 2026
Your Name: Luca Sofia
Affiliation: *Dept. of Health Sciences, University of Genoa*
Manuscript Title: Structural MRI Signature Predicts Tau Staging in Alzheimer's Disease
Manuscript Number: DADM-20260508

Relationship / activity / interest	None	Name all entities (or indicate none)	Specifications / comments
**1.** All support for the present manuscript (e.g., funding, provision of study materials, medical writing, article-processing charges, etc.). [No time limit for this item.]	☒	None	—
**2.** Grants or contracts from any entity (if not indicated in item #1 above).	☒	None	—
**3.** Royalties or licenses	☒	None	—
**4.** Consulting fees	☒	None	—
**5.** Payment or honoraria for lectures, presentations, speakers bureaus, manuscript writing or educational events	☒	None	—
**6.** Payment for expert testimony	☒	None	—
**7.** Support for attending meetings and/or travel	☒	None	—
**8.** Patents planned, issued or pending	☒	None	—
**9.** Participation on a Data Safety Monitoring Board or Advisory Board	☒	None	—
**10.** Leadership or fiduciary role in other board, society, committee or advocacy group, paid or unpaid	☒	None	—
**11.** Stock or stock options	☒	None	—
**12.** Receipt of equipment, materials, drugs, medical writing, gifts or other services	☒	None	—
**13.** Other financial or non-financial interests	☒	None	—

☒ I certify that I have answered every question and have not altered the wording of any of the questions on this form.

Signature: L.S. **Date:** 2nd July 2026

ICMJE Disclosure Form

Date: 2nd July 2026

Your Name: Stefano Raffa

Affiliation: *IRCCS Azienda Ospedaliera Metropolitana (Ospedale Policlinico San Martino), Genoa*

Manuscript Title: Structural MRI Signature Predicts Tau Staging in Alzheimer's Disease

Manuscript Number: DADM-20260508

Relationship / activity / interest	None	Name all entities (or indicate none)	Specifications / comments
**1.** All support for the present manuscript (e.g., funding, provision of study materials, medical writing, article-processing charges, etc.). [No time limit for this item.]	☒	None	—
**2.** Grants or contracts from any entity (if not indicated in item #1 above).	☒	None	—
**3.** Royalties or licenses	☒	None	—
**4.** Consulting fees	☒	None	—
**5.** Payment or honoraria for lectures, presentations, speakers bureaus, manuscript writing or educational events	☒	None	—
**6.** Payment for expert testimony	☒	None	—
**7.** Support for attending meetings and/or travel	☒	None	—
**8.** Patents planned, issued or pending	☒	None	—
**9.** Participation on a Data Safety Monitoring Board or Advisory Board	☒	None	—
**10.** Leadership or fiduciary role in other board, society, committee or advocacy group, paid or unpaid	☒	None	—
**11.** Stock or stock options	☒	None	—
**12.** Receipt of equipment, materials, drugs, medical writing, gifts or other services	☒	None	—
**13.** Other financial or non-financial interests	☒	None	—

☒ I certify that I have answered every question and have not altered the wording of any of the questions on this form.

Signature: S. Raffa **Date:** 2nd July 2026

ICMJE Disclosure Form

Date: 2nd July 2026

Your Name: Gianmario Sambuceti

Affiliation: *IRCCS Azienda Ospedaliera Metropolitana (Ospedale Policlinico San Martino), Genoa; Dept. of Health Sciences, University of Genoa*

Manuscript Title: Structural MRI Signature Predicts Tau Staging in Alzheimer's Disease

Manuscript Number: DADM-20260508

Relationship / activity / interest	None	Name all entities (or indicate none)	Specifications / comments
**1.** All support for the present manuscript (e.g., funding, provision of study materials, medical writing, article-processing charges, etc.). [No time limit for this item.]	☒	None	—
**2.** Grants or contracts from any entity (if not indicated in item #1 above).	☒	None	—
**3.** Royalties or licenses	☒	None	—
**4.** Consulting fees	☒	None	—
**5.** Payment or honoraria for lectures, presentations, speakers bureaus, manuscript writing or educational events	☒	None	—
**6.** Payment for expert testimony	☒	None	—
**7.** Support for attending meetings and/or travel	☒	None	—
**8.** Patents planned, issued or pending	☒	None	—
**9.** Participation on a Data Safety Monitoring Board or Advisory Board	☒	None	—
**10.** Leadership or fiduciary role in other board, society, committee or advocacy group, paid or unpaid	☒	None	—
**11.** Stock or stock options	☒	None	—
**12.** Receipt of equipment, materials, drugs, medical writing, gifts or other services	☒	None	—
**13.** Other financial or non-financial interests	☒	None	—

☒ I certify that I have answered every question and have not altered the wording of any of the questions on this form.

Signature: Sambuceti **Date:** 2nd July 2026

ICMJE Disclosure Form

Date: 2nd July 2026
Your Name: Massimo Caulo
Affiliation: *University G. d'Annunzio of Chieti-Pescara*
Manuscript Title: Structural MRI Signature Predicts Tau Staging in Alzheimer's Disease
Manuscript Number: DADM-20260508

**Relationship / activity / interest**	**None**	**Name all entities (or indicate none)**	**Specifications / comments**
**1.** All support for the present manuscript (e.g., funding, provision of study materials, medical writing, article-processing charges, etc.). [No time limit for this item.]	☒	None	—
**2.** Grants or contracts from any entity (if not indicated in item #1 above).	☒	None	—
**3.** Royalties or licenses	☒	None	—
**4.** Consulting fees	☒	None	—
**5.** Payment or honoraria for lectures, presentations, speakers bureaus, manuscript writing or educational events	☒	None	—
**6.** Payment for expert testimony	☒	None	—
**7.** Support for attending meetings and/or travel	☒	None	—
**8.** Patents planned, issued or pending	☒	None	—
**9.** Participation on a Data Safety Monitoring Board or Advisory Board	☒	None	—
**10.** Leadership or fiduciary role in other board, society, committee or advocacy group, paid or unpaid	☒	None	—
**11.** Stock or stock options	☒	None	—
**12.** Receipt of equipment, materials, drugs, medical writing, gifts or other services	☒	None	—
**13.** Other financial or non-financial interests	☒	None	—

☒ I certify that I have answered every question and have not altered the wording of any of the questions on this form.

Signature: M. Caulo **Date:** 2nd July 2026

ICMJE Disclosure Form

Date: 2nd July 2026

Your Name: Emanuele Pravatà

Affiliation: *University G. d'Annunzio of Chieti-Pescara*

Manuscript Title: Structural MRI Signature Predicts Tau Staging in Alzheimer's Disease

Manuscript Number: DADM-20260508

Relationship / activity / interest	None	Name all entities (or indicate none)	Specifications / comments
**1.** All support for the present manuscript (e.g., funding, provision of study materials, medical writing, article-processing charges, etc.). [No time limit for this item.]	☒	None	—
**2.** Grants or contracts from any entity (if not indicated in item #1 above).	☒	None	—
**3.** Royalties or licenses	☒	None	—
**4.** Consulting fees	☒	None	—
**5.** Payment or honoraria for lectures, presentations, speakers bureaus, manuscript writing or educational events	☒	None	—
**6.** Payment for expert testimony	☒	None	—
**7.** Support for attending meetings and/or travel	☒	None	—
**8.** Patents planned, issued or pending	☒	None	—
**9.** Participation on a Data Safety Monitoring Board or Advisory Board	☒	None	—
**10.** Leadership or fiduciary role in other board, society, committee or advocacy group, paid or unpaid	☒	None	—
**11.** Stock or stock options	☒	None	—
**12.** Receipt of equipment, materials, drugs, medical writing, gifts or other services	☒	None	—
**13.** Other financial or non-financial interests	☒	None	—

☒ I certify that I have answered every question and have not altered the wording of any of the questions on this form.

Signature: Emanuele Pravatà **Date:** 2nd July 2026

ICMJE Disclosure Form

Date: 2nd July 2026

Your Name: Fabrizio Levrero

Affiliation: *IRCCS Azienda Ospedaliera Metropolitana (Ospedale Policlinico San Martino), Genoa*

Manuscript Title: Structural MRI Signature Predicts Tau Staging in Alzheimer's Disease

Manuscript Number: DADM-20260508

Relationship / activity / interest	None	Name all entities (or indicate none)	Specifications / comments
**1.** All support for the present manuscript (e.g., funding, provision of study materials, medical writing, article-processing charges, etc.). [No time limit for this item.]	☒	None	—
**2.** Grants or contracts from any entity (if not indicated in item #1 above).	☒	None	—
**3.** Royalties or licenses	☒	None	—
**4.** Consulting fees	☒	None	—
**5.** Payment or honoraria for lectures, presentations, speakers bureaus, manuscript writing or educational events	☒	None	—
**6.** Payment for expert testimony	☒	None	—
**7.** Support for attending meetings and/or travel	☒	None	—
**8.** Patents planned, issued or pending	☒	None	—
**9.** Participation on a Data Safety Monitoring Board or Advisory Board	☒	None	—
**10.** Leadership or fiduciary role in other board, society, committee or advocacy group, paid or unpaid	☒	None	—
**11.** Stock or stock options	☒	None	—
**12.** Receipt of equipment, materials, drugs, medical writing, gifts or other services	☒	None	—
**13.** Other financial or non-financial interests	☒	None	—

☒ I certify that I have answered every question and have not altered the wording of any of the questions on this form.

Signature: F.L. **Date:** 2nd July 2026

ICMJE Disclosure Form

Date: 2nd July 2026

Your Name: Marco Bozzali

Affiliation: *University of Torino; AOU Città della Salute e della Scienza di Torino*

Manuscript Title: Structural MRI Signature Predicts Tau Staging in Alzheimer's Disease

Manuscript Number: DADM-20260508

Relationship / activity / interest	None	Name all entities (or indicate none)	Specifications / comments
**1.** All support for the present manuscript (e.g., funding, provision of study materials, medical writing, article-processing charges, etc.). [No time limit for this item.]	☒	None	—
**2.** Grants or contracts from any entity (if not indicated in item #1 above).	☒	None	—
**3.** Royalties or licenses	☒	None	—
**4.** Consulting fees	☒	None	—
**5.** Payment or honoraria for lectures, presentations, speakers bureaus, manuscript writing or educational events	☒	None	—
**6.** Payment for expert testimony	☒	None	—
**7.** Support for attending meetings and/or travel	☒	None	—
**8.** Patents planned, issued or pending	☒	None	—
**9.** Participation on a Data Safety Monitoring Board or Advisory Board	☒	None	—
**10.** Leadership or fiduciary role in other board, society, committee or advocacy group, paid or unpaid	☒	None	—
**11.** Stock or stock options	☒	None	—
**12.** Receipt of equipment, materials, drugs, medical writing, gifts or other services	☒	None	—
**13.** Other financial or non-financial interests	☒	None	—

☒ I certify that I have answered every question and have not altered the wording of any of the questions on this form.

Signature: 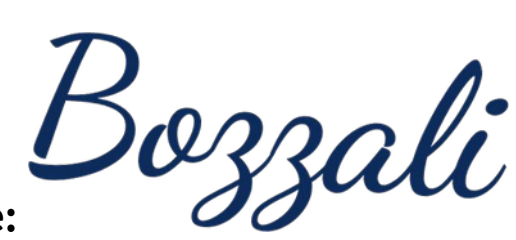

Date: 2nd July 2026

ICMJE Disclosure Form

Date: 2nd July 2026
Your Name: Silvia Morbelli
Affiliation: *AOU Città della Salute e della Scienza di Torino; University of Turin*
Manuscript Title: Structural MRI Signature Predicts Tau Staging in Alzheimer's Disease
Manuscript Number: DADM-20260508

Relationship / activity / interest	None	Name all entities (or indicate none)	Specifications / comments
**1.** All support for the present manuscript (e.g., funding, provision of study materials, medical writing, article-processing charges, etc.). [No time limit for this item.]	☒	None	—
**2.** Grants or contracts from any entity (if not indicated in item #1 above).	☒	None	—
**3.** Royalties or licenses	☒	None	—
**4.** Consulting fees	☒	None	—
**5.** Payment or honoraria for lectures, presentations, speakers bureaus, manuscript writing or educational events	☒	None	—
**6.** Payment for expert testimony	☒	None	—
**7.** Support for attending meetings and/or travel	☒	None	—
**8.** Patents planned, issued or pending	☒	None	—
**9.** Participation on a Data Safety Monitoring Board or Advisory Board	☒	None	—
**10.** Leadership or fiduciary role in other board, society, committee or advocacy group, paid or unpaid	☒	None	—
**11.** Stock or stock options	☒	None	—
**12.** Receipt of equipment, materials, drugs, medical writing, gifts or other services	☒	None	—
**13.** Other financial or non-financial interests	☒	None	—

☒ I certify that I have answered every question and have not altered the wording of any of the questions on this form.

Signature: S. Morbelli **Date:** 2nd July 2026

ICMJE Disclosure Form

Date: 2nd July 2026

Your Name: Michele Piana

Affiliation: *DIMA, University of Genoa; IRCCS Azienda Ospedaliera Metropolitana (Ospedale Policlinico San Martino), Genoa*

Manuscript Title: Structural MRI Signature Predicts Tau Staging in Alzheimer's Disease

Manuscript Number: DADM-20260508

Relationship / activity / interest	None	Name all entities (or indicate none)	Specifications / comments
**1.** All support for the present manuscript (e.g., funding, provision of study materials, medical writing, article-processing charges, etc.). [No time limit for this item.]	☒	None	—
**2.** Grants or contracts from any entity (if not indicated in item #1 above).	☒	None	—
**3.** Royalties or licenses	☒	None	—
**4.** Consulting fees	☒	None	—
**5.** Payment or honoraria for lectures, presentations, speakers bureaus, manuscript writing or educational events	☒	None	—
**6.** Payment for expert testimony	☒	None	—
**7.** Support for attending meetings and/or travel	☒	None	—
**8.** Patents planned, issued or pending	☒	None	—
**9.** Participation on a Data Safety Monitoring Board or Advisory Board	☒	None	—
**10.** Leadership or fiduciary role in other board, society, committee or advocacy group, paid or unpaid	☒	None	—
**11.** Stock or stock options	☒	None	—
**12.** Receipt of equipment, materials, drugs, medical writing, gifts or other services	☒	None	—
**13.** Other financial or non-financial interests	☒	None	—

☒ I certify that I have answered every question and have not altered the wording of any of the questions on this form.

Signature: Michele Piana **Date:** 2nd July 2026

ICMJE Disclosure Form

Date: 2nd July 2026

Your Name: Antonio Uccelli

Affiliation: *DINOGMI, University of Genoa; IRCCS Azienda Ospedaliera Metropolitana (Ospedale Policlinico San Martino), Genoa*

Manuscript Title: Structural MRI Signature Predicts Tau Staging in Alzheimer's Disease

Manuscript Number: DADM-20260508

Relationship / activity / interest	None	Name all entities (or indicate none)	Specifications / comments
**1.** All support for the present manuscript (e.g., funding, provision of study materials, medical writing, article-processing charges, etc.). [No time limit for this item.]	☒	None	—
**2.** Grants or contracts from any entity (if not indicated in item #1 above).	☒	None	—
**3.** Royalties or licenses	☒	None	—
**4.** Consulting fees	☒	None	—
**5.** Payment or honoraria for lectures, presentations, speakers bureaus, manuscript writing or educational events	☒	None	—
**6.** Payment for expert testimony	☒	None	—
**7.** Support for attending meetings and/or travel	☒	None	—
**8.** Patents planned, issued or pending	☒	None	—
**9.** Participation on a Data Safety Monitoring Board or Advisory Board	☒	None	—
**10.** Leadership or fiduciary role in other board, society, committee or advocacy group, paid or unpaid	☒	None	—
**11.** Stock or stock options	☒	None	—
**12.** Receipt of equipment, materials, drugs, medical writing, gifts or other services	☒	None	—
**13.** Other financial or non-financial interests	☒	None	—

☒ I certify that I have answered every question and have not altered the wording of any of the questions on this form.

Signature: A. Uccelli **Date:** 2nd July 2026

ICMJE Disclosure Form

Date: 2nd July 2026

Your Name: Luca Roccatagliata

Affiliation: *IRCCS Azienda Ospedaliera Metropolitana (Ospedale Policlinico San Martino), Genoa; Dept. of Health Sciences, University of Genoa*

Manuscript Title: Structural MRI Signature Predicts Tau Staging in Alzheimer's Disease

Manuscript Number: DADM-20260508

Relationship / activity / interest	None	Name all entities (or indicate none)	Specifications / comments
**1.** All support for the present manuscript (e.g., funding, provision of study materials, medical writing, article-processing charges, etc.). [No time limit for this item.]	☒	None	—
**2.** Grants or contracts from any entity (if not indicated in item #1 above).	☒	None	—
**3.** Royalties or licenses	☒	None	—
**4.** Consulting fees	☒	None	—
**5.** Payment or honoraria for lectures, presentations, speakers bureaus, manuscript writing or educational events	☒	None	—
**6.** Payment for expert testimony	☒	None	—
**7.** Support for attending meetings and/or travel	☒	None	—
**8.** Patents planned, issued or pending	☒	None	—
**9.** Participation on a Data Safety Monitoring Board or Advisory Board	☒	None	—
**10.** Leadership or fiduciary role in other board, society, committee or advocacy group, paid or unpaid	☒	None	—
**11.** Stock or stock options	☒	None	—
**12.** Receipt of equipment, materials, drugs, medical writing, gifts or other services	☒	None	—
**13.** Other financial or non-financial interests	☒	None	—

☒ I certify that I have answered every question and have not altered the wording of any of the questions on this form.

Signature: Roccatagliata **Date:** 2nd July 2026

ICMJE Disclosure Form

Date: 2nd July 2026
Your Name: Andrea Chincarini
Affiliation: *INFN, Genoa*
Manuscript Title: Structural MRI Signature Predicts Tau Staging in Alzheimer's Disease
Manuscript Number: DADM-20260508

Relationship / activity / interest	None	Name all entities (or indicate none)	Specifications / comments
**1.** All support for the present manuscript (e.g., funding, provision of study materials, medical writing, article-processing charges, etc.). [No time limit for this item.]	☒	None	—
**2.** Grants or contracts from any entity (if not indicated in item #1 above).	☒	None	—
**3.** Royalties or licenses	☒	None	—
**4.** Consulting fees	☒	None	—
**5.** Payment or honoraria for lectures, presentations, speakers bureaus, manuscript writing or educational events	☒	None	—
**6.** Payment for expert testimony	☒	None	—
**7.** Support for attending meetings and/or travel	☒	None	—
**8.** Patents planned, issued or pending	☒	None	—
**9.** Participation on a Data Safety Monitoring Board or Advisory Board	☒	None	—
**10.** Leadership or fiduciary role in other board, society, committee or advocacy group, paid or unpaid	☒	None	—
**11.** Stock or stock options	☒	None	—
**12.** Receipt of equipment, materials, drugs, medical writing, gifts or other services	☒	None	—
**13.** Other financial or non-financial interests	☒	None	—

☒ I certify that I have answered every question and have not altered the wording of any of the questions on this form.

Signature: A. Chincarini **Date:** 2nd July 2026

ICMJE Disclosure Form

Date: 2nd July 2026

Your Name: Matteo Pardini (corresponding author)

Affiliation: *DINOGMI, University of Genoa; IRCCS Azienda Ospedaliera Metropolitana (Ospedale Policlinico San Martino), Genoa*

Manuscript Title: Structural MRI Signature Predicts Tau Staging in Alzheimer's Disease

Manuscript Number: DADM-20260508

Relationship / activity / interest	None	Name all entities (or indicate none)	Specifications / comments
**1.** All support for the present manuscript (e.g., funding, provision of study materials, medical writing, article-processing charges, etc.). [No time limit for this item.]	☐	**Italian Ministry of Health / NextGenerationEU – PNRR-MCNT2-2023-12377527 (CUP C13C23001070006)**	Partial support for the present work (project on a clinically feasible multi-parametric quantitative MRI protocol in Alzheimer's disease).
**2.** Grants or contracts from any entity (if not indicated in item #1 above).	☐	**Novartis; Nutricia**	Research support (unrelated to the submitted work).
**3.** Royalties or licenses	☒	None	—
**4.** Consulting fees	☐	**Novartis; Eli Lilly; Eisai; Biogen**	Fees, as reported in the manuscript.
**5.** Payment or honoraria for lectures, presentations, speakers bureaus, manuscript writing or educational events	☒	None	—
**6.** Payment for expert testimony	☒	None	—
**7.** Support for attending meetings and/or travel	☒	None	—
**8.** Patents planned, issued or pending	☒	None	—
**9.** Participation on a Data Safety Monitoring Board or Advisory Board	☒	None	—
**10.** Leadership or fiduciary role in other board, society, committee or advocacy group, paid or unpaid	☒	None	—
**11.** Stock or stock options	☒	None	—
**12.** Receipt of equipment, materials, drugs, medical writing, gifts or other services	☒	None	—
**13.** Other financial or non-financial interests	☒	None	—

☒ I certify that I have answered every question and have not altered the wording of any of the questions on this form.

Signature: Matteo Pardini **Date:** 2nd July 2026
