# Supplementary material for: Structural MRI signature predicts tau staging in Alzheimer's disease
Source: Alzheimers Dement (Amst). 2026 Aug 26;18(3):e70451. doi: 10.1002/dad2.70451 (PMC13508572; doi:10.1002/dad2.70451)
Supplement: Supplementary file 2 [file DAD2-18-e70451-s002.docx]

**Structural MRI signature predicts tau staging in Alzheimer’s Disease**

**Supplementary Materials**

SUPPLEMENTARY TABLE

**Tab.S1** - The table presents the composite ROIs defined by FreeSurfer, including Braak stage-specific ROIs (Braak 1, Braak 3-4, Braak 5-6) and the metatemporal ROI. Each composite ROI consists of multiple brain regions listed in detail within the table.

| **Braak 1** | **Braak 3,4** | **Braak 5,6** | **Metatemporal ROI** |
| --- | --- | --- | --- |
| entorhinal | parahippocampal | superior_frontal | amygdala |
|  | fusiform | lateral_orbitofrontal | entorhinal |
|  | lingual | medial_orbitofrontal | fusiform |
|  | amygdala | frontal_pole | inferiortemporal |
|  | middletemporal | causal_middle_frontal | middletemporal |
|  | caudantcing | rostral_middle_frontal |  |
|  | rostantcing | pars_opercularis |  |
|  | postcing | pars_orbitalis |  |
|  | isthmuscing | pars_triangularis |  |
|  | insula | lateraloccipital |  |
|  | inferiortemporal | parietalsupramarginal |  |
|  | temporalpole | parietalinferior |  |
|  |  | superiortemporal |  |
|  |  | parietalsuperior |  |
|  |  | precuneus |  |
|  |  | bankSuperiorTemporalSulcus |  |
|  |  | tranvtemp |  |
|  |  | pericalcarine |  |
|  |  | postcentral |  |
|  |  | cuneus |  |
|  |  | precentral |  |
|  |  | paracentral |  |

**Tab.S2** - The table reports the outputs (R-squared and p-value) of linear regression models performed using MRI metrics, specifically volume variables.

| **Composite regions of interest** | **Volume variables** | |
| --- | --- | --- |
|  | R-squared | p-value |
| Braak 1 | 0.27 | 2.2e-7 |
| Braak 3,4 | 0.32 | 1.0e-9 |
| Braak 5,6 | 0.35 | 8.0e-12 |
| Metatemporal ROI | 0.33 | 3.5e-10 |
| Global Tau | 0.35 | 1.3e-11 |

SUPPLEMENTARY FIGURES

- **Regional contributions to the MRI tau signature - Cortical thickness variables**

**Fig.S1** - The figure shows the top 20 cortical thickness regions contributing to the prediction of tau loads for the Braak 1 region. The x-axis values represent the distribution of the loadings (weights) corresponding to each covariate within a given model, across all subjects: positive if there is a directly proportional dependence between Braak 1 ROI tau levels and the MRI regional metric; negative if there is an inversely proportional dependence.

**Fig.S2** - The figure shows the top 20 cortical thickness regions contributing to the prediction of tau loads for the Braak 3,4 region. The x-axis values represent the distribution of the loadings (weights) corresponding to each covariate within a given model, across all subjects: positive if there is a directly proportional dependence between Braak 3,4 tau levels and the MRI regional metric; negative if there is an inversely proportional dependence.

**Fig.S3** - The figure shows the top 20 cortical thickness regions contributing to the prediction of tau loads for the Braak 5,6 region. The x-axis values represent the distribution of the loadings (weights) corresponding to each covariate within a given model, across all subjects: positive if there is a directly proportional dependence between Braak 5,6 tau levels and the MRI regional metric; negative if there is an inversely proportional dependence.

**Fig.S4** - The figure shows the top 20 cortical thickness regions contributing to the prediction of tau loads for the Meta-Temporal regions of interest. The x-axis values represent the distribution of the loadings (weights) corresponding to each covariate within a given model, across all subjects: positive if there is a directly proportional dependence between the meta-temporal ROI tau levels and the MRI regional metric; negative if there is an inversely proportional dependence.

**Fig.S5** - The figure shows the top 20 cortical thickness regions contributing to the prediction of tau loads for the Global tau burden. The x-axis values represent the distribution of the loadings (weights) corresponding to each covariate within a given model, across all subjects: positive if there is a directly proportional dependence between global tau levels and the MRI regional metric; negative if there is an inversely proportional dependence.

- **MRI signature classification of tau levels across Braak regions, the metatemporal ROI and the global tau metric**

**Fig.S6 -** Odds (likelihood ratio) distribution according to the composite MRI signature models for each Braak ROIs and for the meta-temporal ROI categorial tau load.

**Fig.S7** - Odds (likelihood ratio) distribution according to the composite MRI signature models for global tau ROI categorial tau load.

- **Alternative normalizations**

**ROI-specific z-score normalization**

**Fig.S8** - The figure shows the MRI signature classification of tau levels in different Braak areas and metatemporal ROI based on ROI-specific z-scores normalization where Clinical (MMSE) and demographic (AGE) variables are included.

**Fig.S9** - The figure shows the MRI signature classification of Global Tau levels in high and low tau burden based on the global metaROI SUVR, based on ROI-specific z-scores normalization where Clinical (MMSE) and demographic (AGE) variables are included.

**Global mean and standard deviation normalization**

**Fig.S10** - The figure shows the MRI signature classification of tau levels in different Braak areas and metatemporal ROI based on global mean and standard deviation normalization where Clinical (MMSE) and demographic (AGE) variables are included.

**Fig.S11** - The figure shows the MRI signature classification of Global Tau levels in high and low tau burden based on the global mean and standard deviation normalization where Clinical (MMSE) and demographic (AGE) variables are included.
